# Supplementary material for: Motivational discourse and campaign-style mobilisation: A positive discourse analysis of language poverty alleviation discourse in China
Source: PLoS One. 2023 Oct 12;18(10):e0292834. doi: 10.1371/journal.pone.0292834 (PMC10569573; doi:10.1371/journal.pone.0292834)
Supplement: Attachment — Submitted filename: RE - Motivational discourse_clean version (1).docx [file pone.0292834.s001.docx]

**A motivational discourse in Chinese language policies: A positive discourse analysis of language poverty alleviation discourse in China**

Run LI,^[1]^ Yating YU,^[2]^* Tayden Fung CHAN^[3]^

**Abstract:**

Many studies have investigated language poverty alleviation in the Chinese context linguistically or socially. However, little research attention has been given to its language policy discourse, which is rather vital because it can be deemed as the preliminary of language poverty alleviation action, directly determining the success of its implementation. To fill this gap, the study employed positive discourse analysis to investigate discursive strategies used in the discourse of language poverty alleviation in Chinese language policies. The finding shows that through four main discursive strategies – nomination, argumentation, perspectivization, and predication, official documents concerning language poverty alleviation constructed a motivational discourse and applied campaign-style mobilisation to encourage people to follow the implementation of language poverty alleviation. This study sheds light on the official purpose of using certain discursive strategies in language poverty alleviation discourse and some ideological implications behind it.

**Keywords:** campaign-style policy implementation, Chinese language policies, language poverty alleviation, motivational discourse, positive discourse analysis

**Introduction**

Poverty appears to be a worldwide phenomenon, afflicting developing countries in particular [1–3]. Poverty has historically been regarded as an unavoidable trend because the low production of non-industrialised economics cannot keep up with the rapidly growing population [4], which leads to “famine, starvation, and deprivation of basic necessities” [5]. As a result, poverty alleviation is urgent in order to lift people out of poverty [6]. There are numerous approaches and perspectives presented to address poverty. Ahmad [7] concentrated on how to deal with water scarcity and unequal water distribution, which cause poverty in some communities. Zuckerman [8] emphasised gender inequality, advocating for women’s empowerment because undergoing different experiences of poverty, women and men “have different poverty reduction priorities and are affected differently by development interventions”. Furthermore, climate change is linked to poverty because the climate has a large impact on agricultural production, which directly concerns people’s welfare [9], and surveys found that both mitigation of and adaptation to climate change are required to avoid any risk that may lead to poverty [10]. Literacy and education should not be overlooked because they play an important role in poverty alleviation [11], and language is an essential component of literacy education [12]. As a result, language poverty alleviation (LPA) has attracted scholars’ attention in recent years [13–16].

LPA is the action that alleviates poverty in specific regions by developing language policy and language planning [16] in order to potentially generate economic capital transferred from linguistic capital [17]. In China, LPA is to popularise Mandarin, particularly in impoverished areas [15], while also preserving and advocating minority languages [18].

LPA has attracted some scholarly attention on a global scale. Igwebuike [19] conducted an LPA study in West Africa. Through the lens of the Millennium Development Goals, he discovered that indigenous West African languages are still important, despite being excluded. Many people are unable to understand some economic intervention schemes related to indigenous languages that prevent poverty due to the use of ex-colonial languages. Nonetheless, language policy is rarely effective in West Africa, and it has no positive impact on indigenous languages, which can generate wealth through some relevant professions such as teachers, translators, and interpreters. Igwebuike [19] emphasises the popularisation of a specific language and advocates that people can seek fortune on their own initiative as a result of their linguistic capital. As a result, effective language policy implementation and micro-language planning would be critical in alleviating poverty in West Africa. Similarly, underscoring the importance of language in alleviating poverty, Abdurraheem and Suraju [20] investigated how linguistic features such as lexical repetition, lexical collocation, synonyms, presuppositions, and implicature, are constructed in Glorious, thereby reflecting the significance of Zakat in promoting poverty alleviation in Nigeria and showcasing the power and function of linguistic features in particular discourse in inspiring people to take poverty alleviation actions.^1^

Despite the fact that there are not many LPA studies in China, they are getting widespread attention. Li et al. [17] investigated the language attitudes of people in impoverished areas using mixed methods (questionnaires, focused interviews, and observation). They discovered that Hani (a minority language in China) would be transferred into economic capital from linguistic capital and it could also be well developed as Mandarin because multilingual products are indeed favoured by consumers, particularly in submarkets such as tourism. Similarly, R. Li [21] investigated the role of the national lingua franca in poverty alleviation in China through a fieldwork study in the Tibetan region of Qinghai province. She discovered that Mandarin, serving as an inter-ethnic lingua franca, helps to break down barriers between minority groups, promotes communication and commercial interaction, and furthers economic development.

Although some studies have shifted their attention to LPA issues both domestically and internationally, few studies linguistically focus on Chinese LPA discourse construction. The investigation in this field is critical because we can detect how the Chinese government formulates a motivational discourse to inspire people and enable them to support certain language policies through discourse analysis of official documents related to language policy. According to Li et al. [17], the popularisation of Mandarin is emphasised by the Chinese government, and the importance of some minority languages does not receive adequate attention. In such a context, how discourse in language policy in LPA convinces and motivates people to support the popularisation of Mandarin is a question that needs to be investigated. To fill this gap, this study proposes three research questions below to investigate discursive strategies employed in official documents regarding LPA in China from the perspective of positive discourse analysis (PDA), and to investigate how these strategies work.

1. What discursive strategies does the government use in language policies regarding LPA?

2. How do these discursive strategies formulate a motivational discourse to accelerate the implementation of LPA policies?

3. What are the ideological implications for using certain discursive strategies?

**Data collection**

LPA did not officially receive adequate attention until it was first discussed in *Study of Language Policies in China* (*语言政策研究报告*), an official language policy document published in 2021. Besides, two other official reports regarding LPA, inextricably entangled with the language policies, are also presented in *Language Development in China* (*中国语言文字事业发展报告*) and *Language Situation in China* (*语言生活状况报告*). Since the aim of this study is to explore how the official discourse of LAP was formed to motivate people to follow the LPA initiative before the official actions of LAP are done, the criteria for article selection are as follows. First, selected articles should represent the official stance. Second, selected articles should serve as the preliminaries to LPA actions. As such, only three articles are suitable to serve the analysis of this study. These articles have special significance and are “highly valued in the community or […] [have] special significance in some domain such as history or politics [and, thus, can be treated] as artefacts – objects of study in their own right” [22]. Additionally, these exceptional single texts can inspire courageous deeds, change the course of history, and give hope in sad times. These articles, gathered from China National Knowledge Infrastructure (CNKI),^2^ are all based on official documents published by the State Language Commission,^3^ and the data from these articles is used to build a corpus for analysis (Table 1).^4^

**Table1.** Information of the selected data

| **Articles** | **Source** | **Word count** |
| --- | --- | --- |
| 语言扶贫  (Language Poverty Alleviation) | 语言政策研究报告  (Study of Language Policies in China: 2021) | 3843 |
| 推普助力脱贫攻坚  (Mandarin Promotion Facilitates Poverty Alleviation) | 中国语言文字事业发展报告  (Language Development in China: 2021) | 3991 |
| 脱贫攻坚收官年的语言扶贫  (Language Poverty Alleviation in the Year of Eradicating Poverty) | 语言生活状况报告  (Language Situation in China: 2021) | 1937 |

**Analytical framework and procedure**

Martin (2004) first proposed positive discourse analysis (PDA), which “functions to make the world a better place” [23]. Unlike critical discourse analysis (CDA), which deconstructs discrimination based on class, ethnicity, gender, and so on as a result of ideology [23], PDA serves as a complementary perspective to CDA, aiming to identify discourse that has the momentum to promote social development and changes [24]. Hughes [25] describes the function of PDA as “enhancing human flourishing and mitigating social ills”, which perfectly echoes the topic of this study and allows us to learn more about how the Chinese government’s discourse on LPA inspires people to advocate language policies of learning Mandarin to contribute to poverty alleviation, a common enemy facing people. In other words, rather than completely deconstructing social practices, PDA tends to construct them [23,26–28].

However, as a newly developed branch, PDA has a limited interrelationship with CDA [24], and the lack of a contextual framework remains the critical weakness of PDA [26]. As a result, from the standpoint of PDA, the authors analysed the data using discursive strategy identification to examine discursive strategies in the LPA discourse because they can be used by the government to construct inspiring information that has the potential to turn social anxiety into optimism [28].

The analysis of this study employs Reisigl and Wodak’s [29] discursive strategies. Discursive strategies are specific practices used to achieve a goal or represent a viewpoint/ideology [19,28,29], and linguistic features should be prioritised when analysing discursive strategies [28]. In this study, discursive strategies refer to the linguistic features used by the Chinese government in official documents regarding LPA, which have the potential to persuade people of the usefulness of LPA language policies and motivate people to learn Mandarin in order to contribute to poverty alleviation in China. Nomination, predication, argumentation, perspectivisation, and intensification/mitigation are the discursive strategies proposed by Wodak [30]. *Nomination* aims to construct social actors or events discursively, whereas *prediction* aims to depict actions or qualifications of them, such as stereotypical/evaluative attributions or traits [30]. *Argumentation* can justify or refute true or false assertions by employing various types of topos. *Perspectivation* represents a point of view through direct or indirect statements, metaphors, and other techniques, with the goal of demonstrating engagement or detachment. Adverbs, hyperboles, and other language elements are used to modify the illocutionary force of utterances as *intensification* or *migration* [30].

We analysed the data through Nvivo 12 Pro software because it allows analysts to develop different coding schemes and do inter-rater reliability tests (Author, 2022). The discursive strategies proposed by Wodak [30] are discussed by two analysts as the analytical framework of the study, after which they analysed the data in the first document (39% of the data). Upon finishing the pilot data analysis, the measurement of inter-rater reliability was applied through the Kappa Coefficient test, which was calculated as 0.88, indicating a high level of consensus [32]. The coding standard was further discussed by three analysts to eliminate the disagreement of a few controversial instances, and the first author then went on to code the rest of the data based on the discussed standard.

**Analysis and findings**

We find that in China, the official LPA discourse is primarily realised through four discursive strategies: referential, predication, argumentation, and perspectivisation. The result is presented in Table 2, and the analysis will mainly focus on the four dominant strategies employed in the LPA discourse.

**Table** 2. Distribution of each discursive strategy contributing to the Chinese LPA discourse

| **Discursive strategies** | **Number of instances/ percentages** |
| --- | --- |
| Nomination | 58 (33%) |
| Argumentation | 46 (26%) |
| Perspectivization | 38 (21%) |
| Predication | 31 (18%) |
| Intensification/mitigation | 4 (2%) |
| ***Total*** | 177 (100%) |

***Nomination***

Nomination is reflected in two ways in the LPA discourse. First, there is a use of metaphor. Poverty is described as a battle throughout the documents, which may foster people’s strong spirit to fight poverty collaboratively [28]. Second, some words or phrases related to the working field help to build a highly efficient and professional image of the government in order to gain people’s trust.

1. 各省市县地方部门也都出台了相关工作方案，为**打赢**推普助力脱贫攻坚提供落实保障。

(Local departments in all provinces, cities and counties have also issued relevant work plans to guarantee the ***triumph*** of poverty alleviation through mandarin promotion.)

2. 以坚定的决心和信心决战决胜脱贫**攻坚**，为助力**打赢**脱贫**攻坚战**、全面建成小康社会奠定良好基础。

(***Win the battle*** against poverty with firm determination and confidence to lay a good foundation for helping ***win the battle*** against poverty and build a moderately prosperous society in an all-around way.)

In Examples 1 and 2, “打赢 (triumph)” and “打赢攻坚战 (win the battle)” demonstrate that poverty is a common enemy that people must all work together to combat. The metaphor vehicle of *WAR* portrays people as active actors, endowing those involved in the “battle” with hero-like qualities that deserve to be praised. As a result, the power of language is fully reflected: people, particularly those who lack confidence or are hopelessly trapped in a poor situation, are encouraged to participate in the battle against poverty with determination to win the battle. Furthermore, Example 1 indicates that “相关工作方案 (relevant work plans)” issued by “各省市县地方部门 (Local departments in all provinces, cities and counties)” guarantee winning the battle. It suggests that the government always takes the lead and bears great responsibility to ensure the victory of the battle. The Chinese leaders in a great war are always featured with “high competence, moral strength, and the ability to inspire and lead men” [33], thereby constructing a positive (strategic and responsible) image of the government, gaining trust and support from people, and also enabling people to be more confident and determined for the government’s solid support. In the context of Examples 1 and 2, the prerequisite for victory is the promotion and popularisation of Mandarin, which is depicted as a good way to combat poverty. People are more likely to follow the LPA policy because the wartime rhetoric can raise people’s morale in the battle and strengthen the unity of people who share common interests with implications for collectivism [24,28].

3. 充分发挥学科优势，大规模、长时间、高质量地开展线上**示范培训**。

(Give full play to the advantages of disciplines, and conduct large-scale, long-term and high-quality online **demonstration training**.)

4. 制定**指标考核体系**，强化对推普助力脱贫攻坚工作的**指导**和**督查。**

(Formulate an **indicator assessment system** to strengthen the **guidance** and **supervision** of poverty alleviation by promoting mandarin.)

5. **专家**与基层扶贫人员结合，**专家**在评价中发挥主体作用。

(**Experts** (should) work with grass-roots officers who are responsible for poverty alleviation, and **experts** (should) play the main role in the evaluation.)

Some professional words from the working field can also be found in the LPA discourse. For instance, Example 3 highlights that the central government does more than just sending commands. It organises “示范培训 (demonstration training)” that are displayed to relevant departments in various regions to ensure the efficient execution of LPA, revealing the high standard that the government expects from Mandarin production. Similarly, “指标考核系统 (indicator assessment system)” in Example 4 motivates relevant officers because their salaries are directly related to their performance, and this systematic approach helps to ensure the successful implementation of LPA. Furthermore, “指导和监督 (guidance and supervision)” in a strict way emphasise the great importance attached to LPA, as well as the government’s resolve to eradicate poverty through Mandarin promotion. Moreover, “专家 (experts)” in Example 5 should not be overlooked. Typically, experts increase the credibility of a specific activity [34], particularly from the perspective of readers who are unfamiliar with LPA in this context, and the professionalism that “experts” bring can win people’s trust. Meanwhile, it persuades people that LPA is on the right track and will be successful in the end. Poverty has a significant impact on all people who are affected by it. Therefore, under the circumstances of poverty, people are in the community with common interests. The emphasis on professionalism prompts people who share common interests to show more trust in leaders who are professional in dealing with difficulties [35].

***Argumentation***

Argumentation strategy is well-used in the LPA discourse to primarily demonstrate the justification of poverty alleviation through promoting Mandarin, which can be roughly classified into three aspects: urgency, professionalism, and benefits.

6. 2018年以来，学界在探讨贫困地区推普方略的同时，深入研究语言与贫困的基础理论问题和重大实践问题，推动“语言扶贫”成为近年来语言政策研究的重要话题。

(Since 2018, while discussing the strategy of promoting mandarin in poverty-stricken areas, academic circles have conducted in-depth research on the basic theoretical issues and major practical issues of language and poverty, and promoted “language poverty alleviation” to become an important topic in language policy research in recent years.**)**

7. 2018-2019年间，我国学者以推普助力脱贫实践为核心，展开了集中的语言扶贫理论研究。

(From 2018 to 2019, Chinese scholars carried out intensive theoretical research on language poverty alleviation with the practice of promoting mandarin to alleviate poverty as the core.)

8. 饶高琦、魏晖从语言资源观角度，将语言扶贫界定为“利用语言资源相对优势获取个体或区域语言能力，进而增加个体收入或促进区域经济社会发展的一种扶贫方式”。

(From the perspective of language resources, Rao Gaoqi, and Wei Hui define language poverty alleviation as “a poverty alleviation method that uses the comparative advantage of language resources to obtain individual or regional language ability, thereby increasing individual income or promoting regional economic and social development”.)

9. 会普通话的贫困户2018年的打工收入比不会普通话的贫困户高32.8%；按照国家3747元农村家庭人均年收入的精准扶贫标准统计，会普通话家庭的脱贫率增加了20%左右。

(In 2018, the income of poor households who can speak Mandarin is 32.8% higher than that of poor households who do not speak Mandarin. According to the country’s targeted poverty alleviation standard of 3,747 yuan per capita annual income for rural households, the poverty alleviation rate of households who speak Mandarin has increased by about 20%.)

Example 6 is a topos of urgency that treats LPA as “基础理论问题和重大实践问题 (basic theoretical issues and major practical issues)”, and “重要话题 (important topic)” particularly “近年来 (in recent years)”. The importance placed on LPA, particularly in official political discourse, has the potential to raise people’s awareness, making them more likely to engage in this issue, particularly among the poor. “Chinese scholars (我国学者)” in Example 7 demonstrates the topos of authority. They have conducted “集中的理论研究 (intensive theoretical research)” on LPA in recent years, which adds to the point of worthiness and importance. The identity of the scholar, combined with the extensive research they have conducted, increases the credibility of the successful implementation of LPA. Furthermore, scholars regard LPA “为核心 (as the core)”, and place a high value on it. This raises people’s awareness of the significance of the LPA issue, which is closely related to their daily lives, echoing the implication conveyed by Example 6. In Example 8, two experts, Rao Gaoqi and Wei Hui, define LPA as “增加个体收入或促进区域经济社会发展 (increasing individual income or promoting regional economic and social development)”. The seemingly authoritative definition explains the advantages of LPA. One could argue that the definition provided by only two experts appears subjective. However, Example 9 reflects a similar message through the topos of numbers, which are more persuasive, as concluded by some empirical studies. As a result, experts’ definitions and evidence from fieldwork research solidly demonstrate the existence of benefits—Mandarin promotion can promote poverty alleviation. People will show more support and trust in the government on this issue once they are convinced that LPA can indeed lift them out of poverty and improve their life quality.

***Perspectivisation***

By directly quoting opinions from the official meeting, documents, and experts, the Chinese government demonstrates the importance and necessity of promoting Mandarin and also shows that some tangible and systemic actions have been taken in this regard. Furthermore, the perspective of protecting minority languages appears to moderate the seemingly arbitrary notion of “Mandarin as the core” and ensure the equity and diversity of language development.

10. 会议把“坚定不移推广普及国家通用语言文字，助力决战决胜脱贫攻坚”列为专项任务。

(A special task that the meeting proposed is that “national lingua franca should unswervingly be promoted and popularised to contribute to the battle of fighting against poverty”.)

11. 5月18日印发《打赢教育脱贫攻坚收官战总攻方案》，明确要求全面加强各级各类学校国家通用语言文字教育…

(The planning for successfully combating poverty through education> was published on 18^th^, May. It explicitly demands that the education for national lingua franca in schools of different levels should be attached more importance.)

12. 王春辉依据语言的性质和功能明确了推普助力脱贫的机理，并在此基础上构建了推普助力脱贫攻坚效果评估的指标体系。

(According to the essence and function of the language, Wang Chunhui confirms the mechanism of poverty alleviation through promoting mandarin, based on which he proposes the index to evaluate poverty alleviation through promoting mandarin.)

The meeting in Example 10 is China’s annual national language meeting. “推广普及国家通用文字 (Promoting and popularising national lingua franca)” is treated as “专项任务 (a special task)”, indicating that the priority has been given to Mandarin promotion to alleviate poverty. This is the most pressing task at the moment, enabling people to understand the importance of learning Mandarin, as it has been emphasised in the official meeting. Example 10 demonstrates the significance of Mandarin promotion. Apart from the meeting mentioned in Example 10, there are some other official documents involved in the LPA discourse that are specifically aimed at promoting Mandarin to fight poverty. The release of these official documents can directly demonstrate the importance that the government currently places on LPA. According to the official document mentioned in Example 11, “国家通用语言文字教育 (education for national lingua franca)” is “明确要求 (explicitly demanded)”, which can be regarded as a mandatory task for students, particularly for those with limited knowledge about Mandarin. The education is provided in “各级各类学校 (schools of different levels)” to demonstrate its necessity, and it also shows the government’s determination to popularise Mandarin in the country. In Example 12, an expert’s wisdom is given. “机理 (mechanism)” and “指标 (index)” are some actions and accomplishments that have been brought to LPA. People can be assured that LPA is dependable for some plans and accomplishments that experts have attained at the moment. Currently, many experts have conducted extensive research on LPA, many of which are also revered in the LPA discourse. In this way, the government is more likely to gain people’s trust because the government seeks advice from experts and develops numerous feasible plans rather than simply posting slogans or pressuring people to learn Mandarin without making any effort, as evidenced in the data. Therefore, the importance and necessity of LPA conveyed by the government are critical for arousing positive public sentiment, thus more easily enacting language policies and conducting language planning.

13. 刘金林、马静认为，兼顾当地少数民族语言的保护和传承，应是下一步语言扶贫需要考虑和解决的核心问题。

(According to Liu Jinlin and Ma Jing, the protection of and support for minority languages should be the core issue that needs to be considered and resolved in the next step.)

The experts in this case also emphasise the importance of developing minority languages. “少数民族语言的保护与传承 (the protection of and support for minority languages)” should be “核心问题 (the core issue)” in the future, indicating that, in addition to Mandarin popularisation, the government recognises the importance of promoting minority languages as they have distinct educational functions to cognitively and affectively nurture people, particularly children from ethnic minority backgrounds in Example 13 [36]. The promotion of minority languages and Mandarin strengthens minority people’s cultural identity [37] and sense of belonging while also accelerating the process of poverty alleviation, which is a desirable scenario for people.

***Predication***

The motivational discourse shows that the government communicates to the public the ongoing progress in LPA and expresses determination and resolution through a prediction strategy.

14. 2020年的相关研究**进一步丰富和发展了**语言扶贫的内涵，详细地阐释了语言的减贫效应。

(The relevant research in 2020 **further enriched and developed** the content of language poverty alleviation and detailedly explained the poverty reduction effect of language.)

15. **坚定不移**推广普及国家通用语言文字，助力决战决胜脱贫攻坚。

(**Unswervingly** promote and popularise national lingua franca, contributing to the decisive battle against poverty alleviation.)

16. …省部**合作共推**、**社会多元参与**的**协同推进**体系。

(A **coordinated** **promotion** system with **provincial cooperation** and socially **multitiered** **participation**.)

In Example 14, “语言扶贫内涵 (the content of language poverty alleviation)” is “丰富和发展 (enriched and developed)” by “相关研究 (relevant research)”, indicating that LPA is in progress and evolving in a positive direction. “进一步 (further)” modifying “丰富和发展 (enriched and developed) shows more improvements have been made to lay the solid foundation of the final success of LPA. The adverb “坚定不移 (unswervingly)” in Example 15 reveals the determination of the government. Despite the many challenges ahead in poverty alleviation due to China’s large population [38], the Chinese government does not flinch, and it contributes fully to “脱贫攻坚 (the decisive battle against poverty alleviation)”. The unwavering attitude can greatly foster people’s collaborative spirit to participate in LPA. As further illustrated in Example 16, “省部合作共推 (provincial cooperation)”, “社会多元参与 (socially multitiered participation)”, and “协同推进 (coordinated promotion)” demonstrate how the government brings together the power and wisdom of all parts of society to engage in LPA, creating a holistic and united impression. It can lead people to consider that they, too, are a part of the society and thus have obligations to contribute to major national issues that are inextricably linked to their welfare, creating a sense of collectivism and unity. Group culture, which promotes the spirit of cooperation and unity, is popular and important in China for instilling a sense of belonging [39]. As a result, fostering collectivism is common in political discourse in China to motivate people.

**Discussion**

Through the lens of positive discourse analysis and with the help of a corpus, this study identifies the discursive strategies employed by the Chinese government in LPA and how these strategies formulated the motivational discourse to inspire people to support language policies with the aim of promoting poverty alleviation. The primary discursive strategy employed by the Chinese government is the use of nomination in describing the action of poverty alleviation as a battle which encourages people with a common interest to fight the enemy together and instils a sense of collectivism into people. The warfare-like metaphors in the official documents created a pressing atmosphere in which the outcomes of the campaigns determine the destiny of the whole nation. With such a strategy, several advantages for policy implementation are found as follows: (1) the government officials are able to put the agenda of fighting poverty as a top priority and allocate resources to achieve the policy outcome; (2) social mobilisation for common goals distracts people’s attention from unsatisfactory performance of the government and enhances the authority of local officials; and (3) those who do not cooperate with the government could be morally criticised as the spoilers of collectivism. Apart from the wartime atmosphere, the nomination strategy also introduces the scientific implementation and evaluation concept reflected by the training and the indicator assessment system. The local officials are required to fulfil the targets set by the upper-level units in order to have better performance appraisals. Nomination then becomes a feasible and actionable way to mobilise people and officials in the campaigns of poverty alleviation.

Another discursive strategy found in the official documents is the use of argumentation for the purpose of justifying the LPA policies and thus creating a positive image of the Chinese government. The nature of professionalism in LPA is demonstrated by quoting research and expert opinions to gain people’s confidence and trust. The statistics data also shows the effectiveness of promoting Mandarin in alleviating poverty and the extent of improved economic status among the designated areas. Through the augmentation strategy, people and officials can understand the reasons for implementing LPA and its expected outcomes. Moreover, argumentation emphasises the urgency to raise awareness of the importance of carrying out LPA. People may establish the thought that if LPA fails to be achieved, individual welfare and social development will be jeopardised. Overall, the argumentation largely enhances the policy legitimacy and persuades the people to accept the correctness set by the official authorities.

Apart from argumentation, perspectivisation is also a discursive strategy used to enhance the legitimacy of LPA and publicise the policy message. Although the documents set Mandarin as the primary official language to be promoted for poverty alleviation, the significance of other minority languages is also emphasised. If Mandarin is solely popularised, people from ethnic minorities are more likely to be alienated. As such, the inclusion of minority languages on the agenda reassures people that their languages and cultures will not be ignored. This practice strengthens people’s willingness to collaborate with the government at this critical juncture. Such a strategy of perspectivisation gives the impression that the government has fully considered various factors before making the decision and undermines the influence of opposing voices against LPA.

As for predication, the discourses used in the documents display the tangible and precise actions which are being and will be taken by relevant departments. On the one hand, predication expresses the determination to implement the LPA policy; on the other hand, it conveys the message that the LPA policy will be maintained for an extended period to achieve lasting outcomes.

**Concluding Remarks**

The previous section addresses and responds to the first two research questions set in this article – the main discursive strategies used by the government to promote LPA include nomination, argumentation, perspectivization, and predication; and through these four strategies, the motivational discourse was formulated by creating a wartime-like environment, enhancing policy legitimacy with experts’ opinions and diverse viewpoints, and providing a foreseeable development path. For the final question regarding ideological implications, two main views are revealed from the formulation of the motivational discourse.

Firstly, the Chinese government tends to launch campaign-style mobilisation to implement social policies from time to time. With a pressing atmosphere, nominating social mobilisation as a kind of military preparation is a way to save the cost of management under a top-down structure. The institutional setting significantly conforms to the centralised control and requires the lower-level units or individuals to follow the mandates from the higher-level systems [40]. Such mobilisation with ideological morality is conducive to collaboration under the fragmented structures of the current regime. The campaign-style implementation sometime serves as an experimental ground for exploring different policy paths. Once the policy outcomes meet the expectations of social development, the overall measures will be maintained and even transplanted to other localities. However, the campaign-style atmosphere for a specific policy cannot last for an extended period. The principles and spirit of policy content may be downgraded to unnecessarily elaborate writing which does not arouse people’s attention. This phenomenon leaves room for future research on wordings used in the local governments’ publicity materials or official documents.

Secondly, the official line set by the Chinese authorities usually promotes a pluralism and inclusive environment. This article shows that even though Mandarin is designated as a primary language in the measure of alleviating poverty, there is also an intention to protect other minority languages. Having said that, since the policy line might not be followed by the units responsible for the implementation all the time, some detailed measures might be overlooked by the front-line officials. Most of the localities could only focus on the primary key performance indicators of the appraisal system designed for public officers and leading officials. Whether the minority language can be preserved under the current LPA practices is also left open for future research.

**Notes**

1. Zakat is a charity scheme which asks every wealthy Muslim to make a donation at the beginning of a lunar year.
2. CNKI (<https://www.cnki.net>) offers comprehensive academic literature, including books, journals, and conference proceedings.
3. State Language Commission is the official institution of language reform in China, which responsibilities are enacting language policies and planning medium-term and long-term language work, etc.
4. The articles are written in Chinese. In order to precisely present analyses and findings, literal translation, which is faithful to the original texts, is used to avoid potential interference of translation. A translator used the back translation technique to double-check the accuracy of the translation.

**References**

[1]	Lehning AJ, Vu CM, Pintak I. Theories of poverty: Findings from textbooks on human behavior and the social environment. J Hum Behav Soc Environ 2007;26:5–19. https://doi.org/10.1080/10911359.2014.843388.

[2]	Deguara A. The faces of the poor: a sociological review in contemporary Europe and Malta 2017:89–128.

[3]	Donaldson M. What is hegemonic masculinity? Structures of oppression, forces for change. Theory Soc 1993;22:643–57.

[4]	Singh S. Micro finance: A conceptual analysis. J Res Bank Financ 2012;2:44–57.

[5]	Apodaca C. Do global strategies for poverty eradication in Sub-Saharan Africa work? An assessment of several international macroeconomic policies. J Third World Stud 2010;27:109–34.

[6]	Lehmann I, Martin A, Fisher JA. Why Should Ecosystem Services Be Governed to Support Poverty Alleviation? Philosophical Perspectives on Positions in the Empirical Literature. Ecol Econ 2018;149:265–73. https://doi.org/https://doi.org/10.1016/j.ecolecon.2018.03.003.

[7]	Ahmad QK. Towards poverty alleviation: The water sector perspectives. Int J Water Resour Dev 2003;19:263–77. https://doi.org/10.1080/0790062032000089356.

[8]	Zuckerman E. Poverty Reduction Strategy Papers and Gender. Backgr. Pap. Conf. Sustain. Poverty Reduct. PRSPs-Challenges Dev. Ctries. Dev. Coop., Berlin: 2002, p. 1–12.

[9]	Slater R, Peskett L, Ludi E, Brown D. Climate change, agricultural policy and poverty reduction-how much do we know? Nat Resour Perspect 2007;109:1–6.

[10]	Eriksen SE, Klein RJ, Ulsrud K, Næss LO, O’Brien K. Climate change adaptation and poverty reduction: Key interactions and critical measures. GECHS Rep 2007;1:1–44.

[11]	Tilak JB. Post-elementary education, poverty and development in India. Int J Educ Dev 2007;27:435–45.

[12]	Dickinson DK, Tabors PO. Beginning literacy with language: Young children learning at home and school. Paul H Brookes Publishing; 2001.

[13]	Mbenzi PA, Ashikuti SN. Uushiindaism as a collective poverty alleviation mechanism and social support. JULACE J Univ Namibia Lang Cent 2018;3:109. https://doi.org/10.32642/julace.v3i1.1378.

[14]	Adeniran A. Towards language planning for poverty alleviation. In: Ndimele O, editor. Linguist. Parad. A Festschrift E. Nolue Emenanjo, M&J Grand Orbit Commnications Ltd., Port Harcourt; 2019, p. 145–53.

[15]	Liu Z. Path and Effectiveness Evaluation of Language Poverty Alleviation — Based on the Practice in Poor Areas. Proc 2020 Int Conf Lang Commun Cult Stud (ICLCCS 2020) 2021;537:101–8. https://doi.org/10.2991/assehr.k.210313.020.

[16]	Igboanusi H. The role of language policy in poverty alleviation in West Africa. Int J Soc Lang 2014:75–90. https://doi.org/10.1515/ijsl-2013-0066.

[17]	Li J, Xu M, Chen J. A Bourdieusian analysis of the multilingualism in a poverty-stricken ethnic minority area: can linguistic capital be transferred to economic capital? J Multiling Multicult Dev 2022;43:122–39. https://doi.org/10.1080/01434632.2020.1733585.

[18]	Wang Y, Phillion JA. Minority language policy and practice in china: The need for multicultural education. Int J Multicult Educ 2009;11:1–14. https://doi.org/10.18251/ijme.v11i1.138.

[19]	Igwebuike EE. Discursive strategies and ideologies in selected newspaper reports on the Nigerian-Cameroonian Bakassi peninsula border conflict. Commun Public 2018;3:151–168. https://doi.org/https://doi.org/10.1177/2057047317748500.

[20]	Abdurraheem H, Suraju SB. Taming poverty in Nigeria: Language, Zakat and national development. Qudus Int J Islam Stud 2018;6:1–23. https://doi.org/10.21043/qijis.v1i1.3278.

[21]	Li R. On common language education value exploration in ethnic poverty-striken area under the backdrop of targeted poverty alleviation—A case study on poverty stricken Tibetan region in Qinghai province. J Res Educ Ethn Minor 2019;30:58–63.

[22]	Matthiessen CMIM. Frequency profiles of some basic grammatical systems: An interim report. In: Hunston S, Thompson G, editors. Syst. corpus Explor. Connect., London: Equinox; 2006, p. 103–142.

[23]	Martin JR. Positive discourse analysis: Solidarity and change. Rev Canar Estud Ingleses 2004;49:179–202.

[24]	Nartey M. Voice, agency and identity: A positive discourse analysis of ‘resistance’ in the rhetoric of Kwame Nkrumah. Lang Intercult Commun 2020;20:193–205. https://doi.org/10.1080/14708477.2020.1722686.

[25]	Hughes JMF. Progressing positive discourse analysis and/in critical discourse studies: Reconstructing resistance through progressive discourse analysis. Rev Commun 2018;18:193–211. https://doi.org/10.1080/15358593.2018.1479880.

[26]	Bartlett T. Hybrid voices and collaborative change: Contextualising positive discourse analysis. New York: Routledge; 2012.

[27]	Bartlett T. Positive discourse analysis. In: Flowerdew J, Richardson JE, editors. Routledge Handb. Crit. discourse Stud., Routledge; 2017, p. 133–47.

[28]	Sultan S, Rapi M. Positive discourse analysis of the Indonesian Government spokesperson’s discursive strategies during the Covid-19 pandemic. GEMA Online® J Lang Stud 2020;20:251–72. https://doi.org/10.17576/gema-2020-2004-14.

[29]	Reisigl M, Wodak R. The discourse-historical approach. In: Reisigl M, Wodak R, editors. Methods Crit. discourse Anal. 2nd ed., London: Sage; 2009, p. 87–121.

[30]	Wodak R. Critical discourse analysis, discourse‐historical approach. In: Tracy K, Ilie C, Sandel T, editors. Int. Encycl. Lang. Soc. Interact., New York, NY: John Wiley & Sons; 2015, p. 1–14. https://doi.org/10.1002/9781118611463/wbielsi116.

[31]	Yu Y, Tian FF. Leftover or individualised? Representations of Chinese single womanhood in Western English-language news media. Asian Stud Rev 2022;00:1–17. https://doi.org/10.1080/10357823.2021.2023095.

[32]	Cohen J. A coefficient of agreement for nominal scales. Educ Psychol Meas 1960;20:37–46.

[33]	Xu L. Metaphor in action in political discourse. Int. Conf. Econ. Soc. Sci. Arts, Educ. Manag. Eng. (ESSAEME 2015), 2015, p. 397–402. https://doi.org/10.46991/afa/2009.5.1-2.072.

[34]	Lachapelle E, Montpetit É, Gauvin JP. Public perceptions of expert credibility on policy issues: The role of expert framing and political worldviews. Policy Stud J 2014;42:674–97.

[35]	Gill RB. Professionalism, advocacy, and credibility: A futile cycle? Hum Dimens Wildl 2001;6:21–32. https://doi.org/10.1080/10871200152668661.

[36]	Feng A, Adamson B. Language policies and sociolinguistic domains in the context of minority groups in China. J Multiling Multicult Dev 2018;39:169–80. https://doi.org/10.1080/01434632.2017.1340478.

[37]	Pandharipande R. Minority matters: issues in minority languages in India. Int J Multicult Soc 2002;4:213–34.

[38]	Liu Y, Liu J, Zhou Y. Spatio-temporal patterns of rural poverty in China and targeted poverty alleviation strategies. J Rural Stud 2017;52:66–75. https://doi.org/https://doi.org/10.1016/j.jrurstud.2017.04.002.

[39]	Zhou KZ, Gao GY, Yang Z, Zhou N. Developing strategic orientation in China: Antecedents and consequences of market and innovation orientations. J Bus Res 2005;58:1049–58. https://doi.org/10.1016/j.jbusres.2004.02.003.

[40]	Cai C, Jiang W, Tang N. Campaign-style crisis regime: How China responded to the shock of COVID-19. Policy Stud 2022;43:599–619. https://doi.org/10.1080/01442872.2021.1883576.

1. Department of Linguistics and English Language, Lancaster University
2. Department of English and Communication, The Hong Kong Polytechnic University

   *tiffany-yyt.yu@connect.polyu.hk
3. School of Graduate Studies, Lingnan University
