# Supplementary material for: Motivational discourse and campaign-style mobilisation: A positive discourse analysis of language poverty alleviation discourse in China
Source: PLoS One. 2023 Oct 12;18(10):e0292834. doi: 10.1371/journal.pone.0292834 (PMC10569573; doi:10.1371/journal.pone.0292834)
Supplement: Attachment — Submitted filename: 005_Response to Reviewers 20230524.docx [file pone.0292834.s002.docx]

| **Editors and reviewers’ comments:** | **Our response:** |
| --- | --- |
| If your minimal data were obtained from a third party (i.e., data not owned or collected by the authors), please explain how others can access or request the specific datasets related to your research. Confirm that others would be able to access or request these data in the same manner as the authors. Please also confirm that the authors did not have any special access or request privileges that others would not have. | We have included the Data Availability Statement in the main article. |
|  |  |
| **Referee: 1** |  |
| 1. This article presents an interesting study about the discursive strategies employed in the discourse of language poverty alleviation in three Chinese policy-related documents. Overall, the research design is original and technically sound. 2. A very critical point to note is that the “motivational discourse” seems to be a key concept that the authors put forward via analysis. If so, it would be very necessary to give a working definition of it. 3. Moreover, perhaps due to the length of the article, the presented examples are individual sentences extracted from the three documents. However, some complicated concepts would be hard to follow without specific contexts (maybe also because they are Chinese specific?), for instance, “indicator assessment system”. Therefore, I would suggest the authors add more explanatory texts to elaborate them in their corresponding paragraph before analysing the strategies.   By the same token, I’d appreciate it if the authors could provide the three articles so that I can fully grasp them.   4. In addition, it would be better if the authors can relate the current study to previous research in the “Discussion” section. Please do consider what this case study may contribute to a broader international academia on discourse studies, policy studies, or other relevant strands. 5. Please double check wording and grammar. I’ve highlighted some in the manuscript. Other comments and minor suggestions were attached in the manuscript. | 1. We are very grateful for Referee 1’s professional comments and dedicated efforts. We are very impressed by the detailed, constructive, and useful comments. 2. “The working definition of motivational discourse in this study is certain types of spoken or written language in materials (e.g., advertisements, business reports, and official documents) that can motivate and mobilise people to make positive social changes in their lives by employing particular discursive strategies. This type of discourse aims to encourage and inspire the audience/reader to take action to achieve a goal that seems unachievable; it can also further particular political or ideological goals and potentially facilitate serious social changes. In this study, the official documents of Chinese language policies regarding LPA are the data under investigation.” 3. The indicator assessment system has been explained in the endnote: “‘指标考核系统 (indicator assessment system)’, in this study, refers to the guidelines and standards set by the Chinese government for local departments’ work on poverty alleviation [40]. The effectiveness of local departments in terms of poverty alleviation can be evaluated using this system”. We have also provided more explanatory texts for terms, such as “示范培训 (demonstration training)”, to elaborate on the contexts in their corresponding paragraph before analysing the strategies.   We have given the sources of the data. The articles are available to the general public in all national libraries of China. It is also available in China National Knowledge Infrastructure (CNKI) with a subscription. Since we do not hold the copyright to reuse third-party material in our article, we do not provide all the data in the appendix. Instead, we have provided all the data in the supplemented document for your reference.  Please see the reference: National Language Working Committee Group. Study of language policy in China: 2021 [Zhongguo yuyan zhengce yan jiu baogao]. Shanghai: The Commercial Press; 2021.   4. We have provided more discussion to engage with the broader international academia on discourse studies and policy studies. We have also emphasised our contribution in the “Conclusion” section. 5. In addition to the wording and grammar issues pointed out by Referee 1, we also sent the paper out for professional proofreading conducted by a native speaker before we re-submitted it. |
| Other comments and minor suggestions were attached in the manuscript:   1) Consider if it would be better to delete the “A”. Incidentally, I am also thinking about another title “Motivational discourse and campaign-style mobilisation: A positive discourse analysis of language poverty alleviation discourse in China”. Would that be more distinct? 2) “Motivational discourse” is a key concept in this article. I suppose this might be a terminological innovation (never seen any research into “motivational discourse” in your review or in other articles). However, after reading this article, I am afraid I have not found any distinct definition of this type of discourse. Since the authors emphasise it in this article, would it be more appropriate to give a working definition of it? 3) The order of authors presented here are different from that indicated in the system. Please double check. 4) Sociologically? 5) I don’t think the two sentences are strong adversative pairs because discourse analysis that this research employs is a sociolinguistic approach, which might be entailed in the “linguistic or sociological” approaches. The authors might mean something like “many studies have investigated XYZ aspects of language poverty alleviation instead of the language policy discourse”? 6) Might be ambiguous. Rephrase.   I suggest hedge here, or show me why is it “directly determining” instead of “determining” or “influencing”?   7) Please reconsider the order of keywords. Perhaps some concepts like “language poverty alleviation” and “motivational discourse” are more essential than others?   May be “mobilisation” as the authors mentioned? This research is not about “policy implementation”.   8) Are there any specific definitions of “poverty alleviation”? 9) This is a focus in this paragraph but seems to be a passing before introducing LPA. I am afraid many details have been given regarding other aspects, such as “gender inequality”, “climate change”, etc. I’d rather suggest more details of “language”, “education” and “poverty”, instead of simply putting something is “important” or “essential”. 10) Repetitive. Try other expressions. 11) I see weak connections between this action and the definition you choose. Would Mandarin popularisation generate any economic capital? If necessary (for international readership), add a footnote to explain Mandarin or Putonghua popularisation. 12) The current literature review is okay but I would recommend a review based on different themes instead of the binary of “globally” and “in China”. In this way, the thesis statement of each paragraph would inform the specific themes and then it would naturally get into your research aims. 13) Check style consistency. 14) Is there any definition of this concept in your article? It is necessary to know “what is a motivational discourse” before we understand how a motivational discourse is formulated. 15) I’m afraid rhetoric seems not appropriate in scholarly articles. 16) Please justify why you choose Reisigl and Wodak’s discursive strategies. Are there any new discursive strategies you might find in addition to those ones? 17) This table presents the distribution of each strategy. However, the numbers are simply put here without any following paragraphs (if there are words or sentences elsewhere that I did not spot, please consider shifting them here) to illustrate the statistics. This table entails some interesting facts that I expect to be elaborated or at least mentioned (for instance, the highest proportion of the use of nomination; the least proportion of intensification/mitigation). I know this article mainly focuses on the qualitative analysis (more specifically, PDA). Since the authors put statistics here, please do consider if it would be more appropriate to add some descriptions (just some may be enough, not too much). 18) Hedge 19) What do you mean by “Mandarin production” here? 20) What does this system refer to? Some concepts like this may need explanation as they seem complicated. They get more complicated as the sub-contexts (in what types of scenarios?) and actors (teachers? officials?) are possibly not the same in those individual examples. 21) This section may be rewritten as I see no dialogue with other scholarly works here in the Discussion section. It looks like “Findings” rather than “Discussion”. It seems to be a recap of the study. This may make your scholarly contribution less explicit. Therefore, I would suggest relating to other relevant research and emphasising the contribution of this study. 22) “used to introduce a statement that is followed by another contrasting statement which is typically introduced with on the other hand” (Merrian-Webster Dictionary) The two clauses are progressive instead of contrastive. 23) I’m a little bit confused. What does the “unnecessarily elaborate writing” refer to? Why won’t they arouse people’s attention? | Thank you once again for providing such valuable and insightful recommendations on our manuscript. We are truly grateful for the time and effort you have invested in helping us enhance the quality of our paper. We hope the revisions meet your expectations and adequately address any concerns you previously had about the paper. We are deeply appreciative of your invaluable guidance and support. Our revision is as follows:   1) “Motivational discourse and campaign-style mobilisation: A positive discourse analysis of language poverty alleviation discourse in China” 2) “The working definition of motivational discourse in this study is certain types of spoken or written language in materials (e.g., advertisements, business reports, and official documents) that can motivate and mobilise people to make positive social changes in their lives by employing particular discursive strategies. This type of discourse aims to encourage and inspire the audience/reader to take action to achieve a goal that seems unachievable; it can also further particular political or ideological goals and potentially facilitate serious social changes. In this study, the official documents of Chinese language policies regarding LPA are the data under investigation.” 3) Thank you very much for noticing this detail. We had this problem due to the system. We had changed the order of the authors. 4) We deleted this sentence. 5) “Many studies have investigated language poverty related to aspects of economic assistance, water scarcity, gender inequality, climate change, etc. However, a focus on language policy discourse has been lacking.” 6) “Language policy discourse is vital because it can be deemed as preliminary to language poverty alleviation action, influencing the success of its implementation.” 7) “Keywords: language poverty alleviation, motivational discourse, campaign-style mobilisation, Chinese language policies, positive discourse analysis” 8) “Poverty alleviation can be seen as a serious action to meet the needs of the poor [7], including productive employment, microfinance, social insurance schemes, social assistance payments, etc. [8]. In addition to economic interventions in poverty alleviation, there are numerous other approaches and perspectives presented to address poverty, such as dealing with gender inequality [9] and climate change [10,11], as well as water scarcity and unequal water distribution [12].” 9) Based on Referees 1’s and 2’s suggestions, we have systematically restructured the section “Introduction” based on different themes. We have also pointed out the shortcomings of their studies, which led to our research aims. 10) “Subsequently” 11) “LPA is an action that alleviates poverty in specific regions by developing language policy and language planning [18] in order to potentially generate economic capital transferred from linguistic capital [19]. In China, the focus of LPA is to popularise Mandarin, particularly in impoverished areas [17], because the popularisation of Mandarin can promote the process of neoliberalisation associated with free-market capitalism, which further contributes to economic development [20] while also preserving and advocating minority languages [21] to ensure the flourishing of minority culture in China. The role language development plays in poverty alleviation cannot be underestimated.” 12) Based on Referees 1’s and 2’s suggestions, we have systematically restructured the “Introduction” section based on different themes. We have also pointed out the shortcomings of their studies, which led to our research aims. 13) Li [24] 14) “The working definition of motivational discourse in this study is certain types of spoken or written language in materials (e.g., advertisements, business reports, and official documents) that can motivate and mobilise people to make positive social changes in their lives by employing particular discursive strategies. This type of discourse aims to encourage and inspire the audience/reader to take action to achieve a goal that seems unachievable; it can also further particular political or ideological goals and potentially facilitate serious social changes. In this study, the official documents of Chinese language policies regarding LPA are the data under investigation.” 15) We deleted the phrase. 16) “Reisigl and Wodak’s discursive strategies are suitable for the analysis of this study because they make an effort to take into account existing knowledge of historical sources as well as the social and political environment in which discursive ‘events’ are situated. Nomination, predication, argumentation, perspectivisation, and intensification/mitigation are the discursive strategies proposed by Wodak [36], and they suffice for the analysis of this study.” 17) “The highest proportion of the use is nomination, and the least proportion is intensification/mitigation. The following analysis will mainly focus on the four dominant strategies employed in the LPA discourse.” 18) We deleted this clause. 19) “…Mandarin teaching (to the poor).” 20) The indicator assessment system has been explained in the endnote: “‘指标考核系统 (indicator assessment system)’, in this study, refers to the guidelines and standards set by the Chinese government for local departments’ work on poverty alleviation [40]. The effectiveness of local departments in terms of poverty alleviation can be evaluated using this system”. We have also provided more explanatory texts to elaborate on the contexts in their corresponding paragraph before analysing the strategies.” 21) We have revised the “Discussion” section relating to other relevant research. The “Discussion” section is now an interpretation of analytical findings. We have also emphasised our contribution in the “Conclusion” section. 22) We deleted “On the one hand” and “On the other hand”. 23) We deleted this sentence. |
|  |  |
| **Referee: 2** |  |
| 1. This study employed positive discourse analysis to investigate discursive strategies used in the discourse of language poverty alleviation in Chinese language policies and brought out policy suggestions. However, the argument in the whole article is superficial, and the logic is not perfect enough, so it has not reached the publication standard of the journal. 2. In Introduction, many related researches have been introduced yet in a simple listing way without systematic generalisations or comments concerning each category. 3. The reports mentioned in Data collection should be given the sources. 4. It would be better if the authors can use a diagram to show the analytical framework and procedure of this study. 5. The corpus used in this study has a relatively small scale, with only 177 cases discovered, which is insufficient to draw a conclusion. 6. In every section of strategy analysis, the authors only gave several examples and shortly explained them. Even in Discussion, the analytical work is still not profound enough. However, we hope to see deeper and more theoretical analysis of each strategy, and more in a logical way, e. g. subsuming some patterns. 7. In Concluding remarks, the points of view brought out lack solid background, and it’s hard to tell how the authors reach these conclusions. 8. The English used in this paper can be further polished to reach academic standard. 9. The literatures cited in this paper failed to reflect the cutting-edge researches in this field in recent years. | 1. We value Referee 2’s comment very highly. We have highlighted the changes we have made in the manuscript. 2. Based on Referees 2’s and 1’s suggestions, we have systematically restructured the “Introduction” section based on different themes. We have also pointed out the shortcomings of their studies, which led to our research aims. 3. We have given the sources of the data. The data is available to the general public in all national libraries of China. It is also available in China National Knowledge Infrastructure (CNKI) with a subscription. Since we do not hold the copyright to reuse third-party material in our article, we do not provide all the data in the appendix. Instead, we have provided all the data in the supplemented document for your reference.   Please see the reference: National Language Working Committee Group. Study of language policy in China: 2021 [Zhongguo yuyan zhengce yan jiu baogao]. Shanghai: The Commercial Press; 2021.   4. We have provided Figures 1 and 2 to show the analytical framework and procedure of this study. 5. The dataset size is subject to the given research objectives. “This study used purposive sampling, which considers the whole data set of interest, and generalisations are limited to the policy documents analysed. Since the aim of this study is to explore how the official discourse of LAP was formed to motivate people to follow the LPA initiative before the official actions of LAP are implemented, the criteria for article selection are” stated in the manuscript. As such, for the purpose of exploring language policy discourse in China, “the official documents of Chinese language policies regarding LPA are the data under investigation”.   In critical discourse studies, despite the dataset size, the selected data should “have special significance and are ‘highly valued in the community or […] [have] special significance in some domain such as history or politics [and, thus, can be treated] as artefacts – objects of study in their own right’ [25]. Additionally, exceptional single texts can inspire courageous deeds, change the course of history, and give hope in sad times”.  The dataset size of our collected articles has 21,347 words, which is not small compared with other critical discourse studies, such as Mwinlaaru and Nartey (2022). Their dataset size has only “1, 976 running words.” (Mwinlaaru & Nartey, 2022, p. 558).   6. We have provided more explanatory texts to elaborate on the contexts in their corresponding paragraph before analysing the strategies in the “Results” section. We have also revised the “Discussion” section relating to other relevant scholarly works. The “Discussion” section is now an interpretation of analytical findings. In addition, we have emphasised our contribution in the “Conclusion” section. 7. We have revised the “Discussion” and “Conclusion” sections. The “Discussion” section is now an interpretation of analytical findings, relating to other relevant research. The conclusion is a summary of what we have done in the study and the contribution of this study. 8. In addition to the wording and grammar issues pointed out by Referee 1, we also sent the paper out for professional proofreading conducted by a native speaker before we re-submitted it. 9. We have inserted an updated literature review.   Once again, thank you for your insightful and helpful comments on our paper. We sincerely appreciate the time and effort you spent assisting us in improving the quality of our paper. We hope the changes live up to your expectations and appropriately resolve any issues you had with the paper in the past. We are incredibly grateful for your helpful advice and assistance. |
