# Supplementary material for: Motivational discourse and campaign-style mobilisation: A positive discourse analysis of language poverty alleviation discourse in China
Source: PLoS One. 2023 Oct 12;18(10):e0292834. doi: 10.1371/journal.pone.0292834 (PMC10569573; doi:10.1371/journal.pone.0292834)
Supplement: Attachment — Submitted filename: Our response.docx [file pone.0292834.s003.docx]

| **Comments of Reviewer #2** | **Our response** |
| --- | --- |
| 1. The main researching part of this article only briefly lists examples and characteristics of four categories, requiring more in-depth theoretical exploration and more innovative discoveries.  2. There are some viewpoints in the Discussion section of the article that lack supporting evidence and are rather casual, such as' fragmented structures of the current region '. There are still many conclusions that come from previous literature or subjective imagination, which cannot be directly derived from the analyzing results of this article, such as “the campaign style atmosphere for a specific policy right not last for an extended period. The principles and spirit of policy content may be downgraded to bureaucratic red tape”. | We would like to express our gratitude for your valuable feedback and constructive comments on our article. We have carefully considered your suggestions and have made significant revisions to address the concerns raised.   1. Regarding your comment about the main research part of the article, we acknowledge the need for more in-depth theoretical exploration and innovative discoveries. In response, we have revised the section to provide a more comprehensive theoretical framework for the concept of campaign-style mobilisation. We have expanded upon the theoretical concepts, specially the LPA motivational discourse and campaign-style mobilisation, in the introduction section, offering a clearer explanation of its key principles and relevance within the broader research context.   Furthermore, we have made substantial efforts to enhance the depth of our theoretical exploration throughout the paper. We have delved into the intricacies of each category by providing detailed analysis and theoretical insights. By incorporating relevant literature and empirical evidence, we have strengthened the theoretical foundation of our research, offering a more robust understanding of campaign-style mobilisation.  In addition, we have introduced innovative discoveries within the paper. We have included fresh perspectives and novel insights that contribute to the existing body of knowledge on the subject matter. In our study, we highlight that the LPA motivational discourse serves as a prime illustration of campaign-style mobilisation and sets an example for other countries/regions as to the popularisation of an official language to tackle poverty alleviation from a linguistic perspective. Our aim has been to present original findings and offers unique interpretations that further advance the field.   2. Firstly, we have addressed the issue of unsupported viewpoints in the Discussion section by deleting the sentences that lack supporting evidence. We understand the importance of presenting a well-grounded argument and have taken steps to ensure that the article maintains a rigorous and evidence-based approach.   Secondly, we have taken your feedback into account and expanded the discussion in the “Discussion” section. We have conducted a more thorough analysis and supported our arguments with relevant evidence. By doing so, we aim to provide a more comprehensive and substantiated discussion of the topic at hand.  We acknowledge that some of the conclusions drawn in the original manuscript were based on previous literature or subjective imagination, rather than directly derived from the analysing results of our study. To rectify this, we have revised the manuscript to ensure that our discussion is firmly rooted in our own analysis and findings.  We believe these revisions have significantly addressed the concerns raised by you and have improved the overall quality of our article. We hope that these changes adequately address your comments and contribute to the advancement of the scholarly discourse on campaign-style mobilisation.  Thank you for your time and consideration. |
